# The Secret Ingredient for Social Success of Young Males: A Functional Polymorphism in the *5HT_2A_* Serotonin Receptor Gene

**DOI:** 10.1371/journal.pone.0054821

**Published:** 2013-02-14

**Authors:** Jan Kornelis Dijkstra, Siegwart Lindenberg, Lieuwe Zijlstra, Esther Bouma, René Veenstra

**Affiliations:** 1 Department of Sociology and Interuniversity Center for Social Science Theory and Methodology (ICS), University of Groningen, Groningen, The Netherlands; 2 Tilburg School of Social and Behavioral Sciences and Tilburg Institute for Behavioral Economics Research (TIBER), Tilburg University, Tilburg, The Netherlands; 3 Department of Sociology, University of Groningen, Groningen, The Netherlands; 4 Faculty of Mathematics and Natural Science, University of Groningen, Groningen, The Netherlands; Radboud University, The Netherlands

## Abstract

In adolescence, being socially successful depends to a large extent on being popular with peers. Even though some youths have what it takes to be popular, they are not, whereas others seem to have a secret ingredient that just makes the difference. In this study the G-allele of a functional polymorphism in the promotor region of the *5HT_2A_* serotonin receptor gene (-G1438A) was identified as a secret ingredient for popularity among peers. These findings build on and extend previous work by Burt (2008, 2009). Tackling limitations from previous research, the role of the *5HT_2A_* serotonin receptor gene was examined in adolescent males (*N* = 285; average age 13) using a unique sample of the TRAILS study. Carrying the G-allele enhanced the relation between aggression and popularity, particularly for those boys who have many female friends. This seems to be an “enhancer” effect of the G-allele whereby popularity relevant characteristics are made more noticeable. There is no “popularity gene”, as the G-allele by itself had no effect on popularity.

## Introduction

What is it that makes some male youths socially successful and others not? Much has been written to answer this question and it basically comes down to the following: in order to be popular among adolescent peers, one must both be aggressive and have some socially positive features [1], [2]. Yet, some male adolescents have all these characteristics and are still not particularly popular, whereas others seem to have something extra, an X-factor that makes all the difference. What might this X-factor be?

In the search for this secret ingredient, we started with a genetic factor that has been found to facilitate social success. In this respect, the serotonin system is a likely candidate since alterations in serotoninergic neurotransmission in the brain have been related to both increased dominance and increased social affiliative behaviors in human [3], [4] and non-human males [5]. A further lead was more specific. In a pioneering study on 202 adolescent boys, Burt [6] found an association between being liked by peers and a genetic polymorphism in the *5HT_2A_* serotonin receptor gene (-G1438A). The AA genotype was weakly related to likeability whereas the GG genotype was more strongly related with the AG genotype in between. In addition, she found that the association between genotype and likeability was partially explained by what she called “rule-breaking behavior” of participants [7]. Burt’s findings are an important indication that it might be worthwhile to focus on adolescent males and on the G-allele of this SNP in the 5HTR_2A_ receptor gene as a possible candidate for the secret ingredient of their social success.

Burt’s research provides an important lead but was limited to experimental situations with only fleeting encounters and no distinct measure of popularity. We know from other research, for example, that being socially successful (i.e. popular) in first encounters is often based on bragging behavior and that this success might not last [8], [9]. Particularly, what is needed but not yet present in Burt’s (2009) experiments are measures of characteristics that are relevant to social success and with which the *5HT_2A_* receptor gene might interact to produce the popularity result.

Generally, people let themselves be influenced by clearly noticeable cues in the present situation [10], [11]. For youths who have popularity-relevant characteristics, the 5HT_2A_ G-allele may act so as to turn these characteristics into better noticeable cues. This fits with Burt’s finding that making suggestions and encouraging peers (ways to become noticeable and the basis for what Burt called “rule-breaking” measures) was positively influenced by the G-allele of the *5HT_2A_* gene. If true, the G-allele of the *5HT_2A_* receptor gene would not be a popularity gene itself but “enhance” popularity-relevant characteristics by making them more noticeable.

What characteristics directly contribute to popularity of males such that their effect can be enhanced by genetic disposition? It is known that aggression contributes to popularity among male adolescents [12], but it has also been found that its effect on popularity is much stronger if it goes along with socially valued features [2], [13]. Affiliative support from girls is an indication that a boy has such socially positive features, and it has been shown to increase the popularity of males in the entire group [14], also among vervet monkeys [5]. Thus, in addition to aggression, we included this support from female friends as a factor that is relevant to popularity for male adolescents.

**Figure 1:**
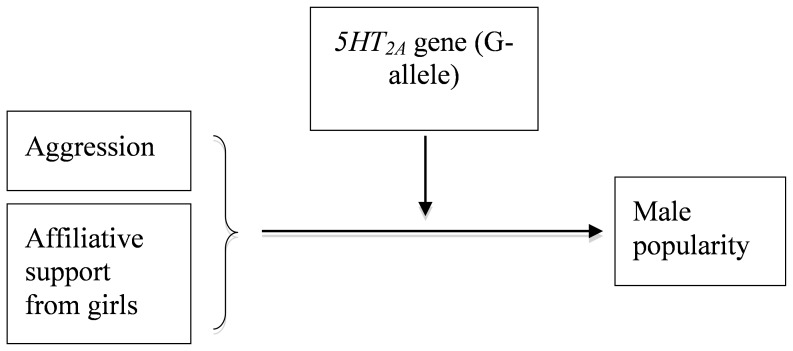
Conceptual model of genetic influence on popularity.

In order to avoid the pitfalls of fleeting encounters, it is necessary to test the conjectures on the role of the -G1438A SNP in the *5HT_2A_* gene in a real-life context with lasting social relations and adequate information on aspects relevant for popularity. Our hypothesis was that the AA, AG, and GG genotypes would not relate to popularity by themselves. Rather we expected that they would enhance the effects of characteristics that directly contribute to popularity. Thus, we expected that aggression and having female friends contribute the most to popularity for boys with the G-allele (see Figure 1).

## Methods

We used data from a large Dutch cohort study, TRAILS (TRacking Adolescents’ Individual Lives Survey), using information from the TRAILS participants [15], [16], and peer nominations from classmates in school classes [2]. To our knowledge, this is the only study to date that contains all the information for the relevant variables and is thus uniquely suited to test our hypotheses. The target sample consisted of 285 Caucasian males (*M* age 13.46, *SD* = 0.52), for whom both genetic information and peer data were available. Genotype was analyzed using a method similar to Burt’s (6), AA (*N* = 50; 17.5%) vs. AG (*N* = 124; 43.5%) vs. GG (*N* = 111; 38.9%). The allele frequencies did not deviate from the Hardy-Weinberg equilibrium (HWE) (see Text S1).

*Popularity* was determined by the proportion of nominations adolescents received from their classmates on the question “Who do others want to be associated with?”, reflecting a reputation-based measure of adolescents who have power to attract peers and are highly desirable as affiliation partners [1]. The scores ranged from 0 to 1, *M*(*SD*) = .10 (.13). *Aggressive behavior* was measured using seven items from the YSR with reasonable internal consistency (α = .72) [17]. Sample items were “I fight a lot”, “I am mean to others”, and “I threaten to hurt people”. Item responses, that is, never (0), sometimes (1), and often (2), were summed and divided by the number of items, *M*(*SD*) = 1.51 (0.39) (*17*). *Female Friends* was measured by summing up and, subsequently, standardizing the nominations male adolescents received from female classmates on the question “Who are your best friends”, *M*(*SD*) = .07 (.11).

## Results

**Figure 2:**
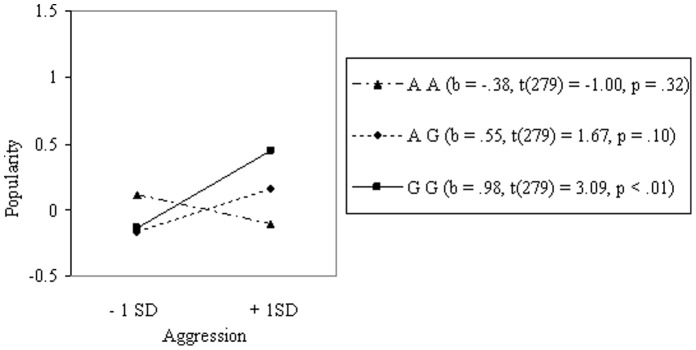
Interaction between Genotype and Aggression on Popularity in Boys (N = 285).

**Figure 3:**
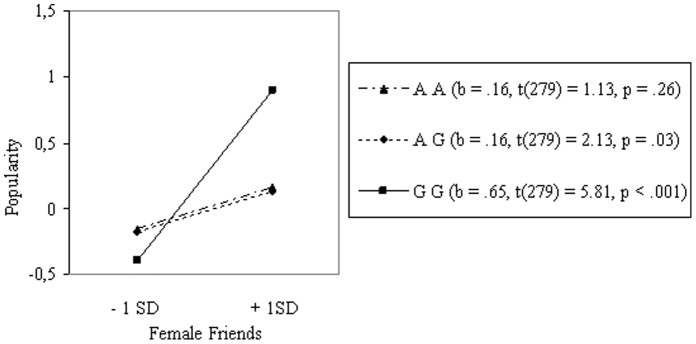
Interaction between Genotype and Having Female Friendships on Popularity in Boys (N = 285).

Aggression (*r* = .14, *p*<.05) and having female friends (*r = *.27, *p*<.001) were both related to popularity (see Table S1 in Text S1). As expected, no mean differences were found directly in aggression or in female friends for genotype (see Table S2 in Text S1). Results of the multiple regression analyses showed that the 5HT_2A_ gene did moderate the relation between aggression and popularity (see Table S3 in Text S1). Using one *SD* above and below the mean as high and low levels for aggression, we calculated the simple slopes for the three different genotypes [18] (see Figure 2). As can be seen, aggression was most strongly associated with popularity for carriers of the GG allele. The same was found for the relation between having female friends and popularity (see Figure 3). In both cases, the G-allele was associated with a larger effect on popularity [12] (see Table S4 in Text S1).

**Figure 4:**
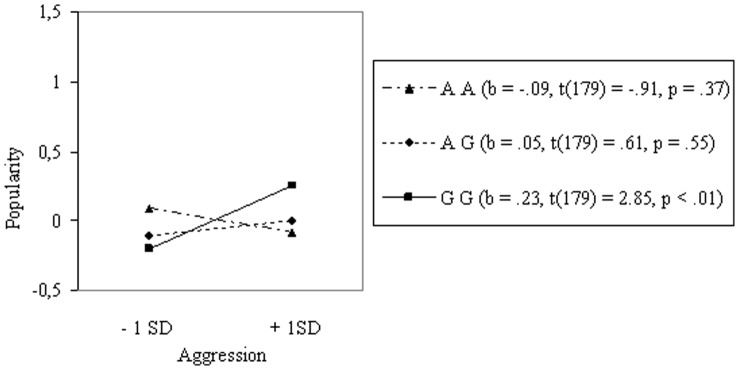
Interaction between Genotype and Aggression on Popularity in Boys with Low Number of Female Friends (N = 185).

**Figure 5:**
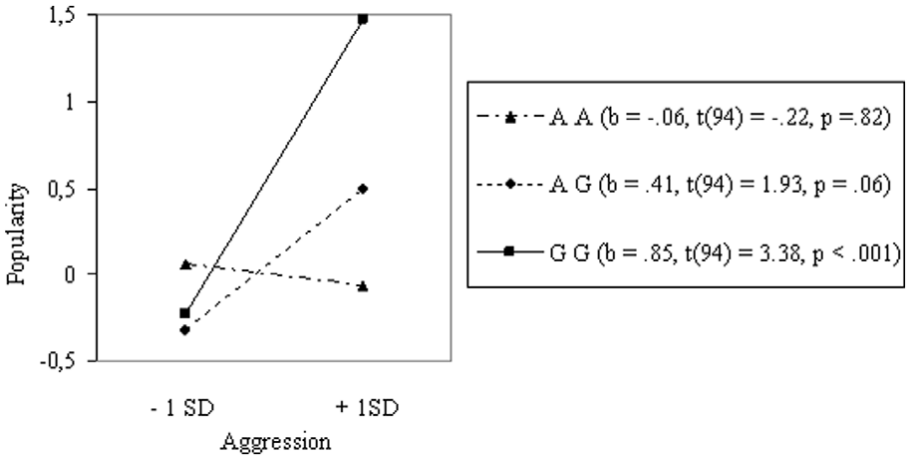
Interaction between Genotype and Aggression on Popularity in Boys with High Number of Female Friends (N = 100).

We then tested the association between aggression and popularity for boys with a low number of female friends (*N* = 185) versus boys with a high number of female friends (*N* = 100) (see Table S3 in Text S1). In Figure 4, we see that for boys with a low number of female friends, genotype moderated the relation between aggression and popularity in the predicted direction, increasing from the AA genotype through the AG to the GG genotype. However, as expected, these effects were much stronger for boys who had a high number of female friends. For these boys, the observed effect of aggression on popularity shoots up from the AA to the GG genotype (see Figure 5).

To give a better impression of the magnitude of these effects, we calculated odds ratios. For boys who have many female friends but also the AA genotype, aggressive behavior does not relate to their popularity at all. By contrast, boys with female friends and the GG genotype seem to use their aggressiveness to their advantage for increasing their popularity. They are more than twice as likely (*OR* = 2.34) to be popular than boys who are one standard deviation lower on aggression. The effect for the AG genotype is in between (*OR* = 1.51). In short, the G-allele truly seems a secret ingredient for the social success of aggressive adolescent boys, especially those with many female friends.

## Discussion

The findings of this study show that the functional SNP in the promotor region of the 5HT_2A_ serotonin receptor gene indeed contains a “secret ingredient” for the social success of adolescent males. Carrying a G-allele considerably enhanced the positive effect of aggression and having female friends on being popular. Burt’s (2009) finding that for boys the G allele increased drawing attention to themselves by making suggestions and encouraging other peers in rule-breaking contexts can be taken as an extra support of this “enhancer” hypothesis. As shown in more detail in the supporting material, the enhancer effect of the allele is also supported by our finding that the allele can even work against social success by making unattractive features more noticeable. Peers who lack prosocial characteristics but have the G allele are particularly low on likeability (see Figure S1).

Similar to Burt (2008; 2009) we explicitly focused on male adolescents. However, we additionally examined to what extent the findings from our study also hold for female adolescents. It appeared that none of the interaction effects were found for girls, suggesting different processes accounting for social success of males and females in the peer group.

This study builds on previous research on the link between the G-allele of the 5HT_2A_ serotonin receptor gene and popularity [6], [7]. However, to our knowledge, this study is the first to show that for established popularity such an effect of the G-allele only occurs in interaction with other variables. The G-allele is a secret ingredient in the social success of aggressive adolescent boys, particularly those with many female friends, but it is not per se a “popularity gene”. Although Burt [6], [7] found a main effect of the G-allele on popularity, she worked with fleeting relationships in experiments. Her finding makes sense if one considers that the G-allele facilitates making an impression with bragging in such fleeting relations with unknown others (as in her study). In our study with lasting relationships and established hierarchies halfway through the school year, not first impressions but bringing certain characteristics (such as aggression) and achievements (such as being liked by girls) to the attention of others will make one popular. This means that in lasting relationships, the G-allele would work mainly in interaction with characteristics that are relevant for popularity. The test of this interactive enhancer effect was possible due to a unique sample that contained a rare combination of data: both genetic data and measures of aggression, being liked by female classmates, and established popularity. Our findings for boys with the GG-allele fit within the differential susceptibility model [19] by showing that particularly this group is susceptible to being either low or high in status, depending in their behaviors. To our knowledge, replication of our findings in another sample is currently not possible, as we know of no other sample that contains all the relevant variables. However, since we built on and extended previous findings, it is unlikely that our findings are simply driven by chance.

With regard to interactive effect of the G-allele with female friends on popularity, we see comparable results for animal studies. Research on male vervet monkeys showed that a dominant position of males strongly depends on the ability to establish positive relationships with females, who even support dominant males in aggressive encounters once the status hierarchy has been established [5]. Thus, overall, our findings fit nicely within previous research on the role of genes in social success [6], [7] and the role of females in social success [5], [14]. Together these findings form a coherent account of genetic factors in popularity.

## Supporting Information

Figure S1 **Interaction between Genotype and Prosocial Behavior on Likeability (N  = 285).**
(TIF)

Text S1 **Supplementary Material - The secret ingredient for the social success.**
(DOC)
