# Supplementary material for: The Secret Ingredient for Social Success of Young Males: A Functional Polymorphism in the 5HT2A Serotonin Receptor Gene
Source: PLoS One. 2013 Feb 14;8(2):e54821. doi: 10.1371/journal.pone.0054821 (PMC3573014; doi:10.1371/journal.pone.0054821)
Supplement: Text S1 — Supplementary Material - The secret ingredient for the social success. (DOC) [file pone.0054821.s002.doc]

Supplementary Material - The secret ingredient for the social success

*Participants and Procedure*

The TRAILS sample originally involved all 10- to 11-year-old children living in five municipalities in the North of the Netherlands, including both urban and rural areas. At T1 2230 participants were enrolled in the study (*M*age = 11.09, *SD* = 0.55, 50.8% girls). At T2, 96.4% of the participants (*N*=2149, *M*age = 13.56; *SD* = 0.53, 51.0% girls) were re-assessed. The response rate at T3 was 81.4% (*N* = 1816, *M*age = 16.28, SD = 0.71, 52.1 % girls). More details on the sampling procedure of the TRAILS sample can be found in De Winter et al. and Huisman et al.[1, 2].

In addition to the regular data collection, peer nominations were collected at T2 in a subsample of TRAILS participants and their classmates. This subsample consisted of 1007 regular TRAILS participants and 2305 of their classmates. Peer nominations were collected in classes with at least three TRAILS participants [3, 4]. The schools provided the names of the classmates. All eligible students then received an information letter for themselves and their parents, in which they were asked to participate. If students or their parents wished to refrain from participation, they were requested to send a reply card within 10 days. In total, 98 students, of whom three TRAILS participants, refused to participate. Approximately two weeks after the information letter was sent, a TRAILS staff member visited the selected school classes to collect the peer nomination data. Because in the first and second grades of Dutch secondary schools adolescents spend most of their time in the same school class, the peer nominations were collected within classrooms. The sociometric data collection lasted about 15 minutes and took place during regular classroom sessions. In the instructions, the staff member emphasized that the data were confidential and that students were not allowed to talk during the test. Students then received the nominations questionnaire and a roster containing the names of all participating classmates. The teacher remained in the classroom during the sociometric administration. The staff member made sure that participants could fill in the questionnaire quietly.

Peer nominations were assessed in 172 classes in 34 schools in the first grade (72 classrooms) and second grade (100 classrooms) of secondary education. The classrooms were evenly divided by educational track: low (60 classrooms), middle (53 classrooms), and high (59 classrooms). Of all 3,672 children that were approached to participate, 90.2% completed the peer nomination measure. This yielded a total of 3,312 students (mean age = 13.60; SD = .66 with 92.7% of the participants aged 12 -14, 1,675 boys, 1,637 girls), including 1,007 regular TRAILS participants (490 boys, 517 girls). Each classroom contained on average 18.39 participants (*SD* = 5.99; range 7 to 30). The ethnic composition of the subsample was 87.3% Caucasian, 0.5% Turkish, 0.6% Moroccan, 1.7% Surinamese, 1.2% Antillian/Aruban, 2.5% Indonesian, 4.1% other, and 2% unknown. Of the 449 Caucasian males for whom peer data and self-reported aggression were available, we had genetic information of 285 persons (*M* age 13.46, *SD* = 0.52).

*Genotyping of the -G1438A SNP (rs6311) in the 5HT*_2A_ *Serotonin Receptor Gene*

DNA was extracted from blood samples (*n*=241) and buccal swabs (*n*=44) (Cytobrush®) at T3, using the manual salting out procedure described by Miller and colleagues (5). SNPs were genotyped at the Genome Analysis Facility, Department of Genetics, University Medical Centre Groningen, University of Groningen, the Netherlands. Genotyping was done on the Golden Gate Illumina BeadStation 500 platform (Illumina Inc., San Diego, CA, USA) following the manufacturer’s protocol and performed by laboratory personnel blinded to the identity of the individual samples. Clustering clouds were manually investigated and adjusted if necessary. Call rate was 99.4% for the A/G SNP (*-G1438A* also known as rs6311). Concordance between DNA replicates showed a genotyping accuracy of 100%. Data cleaning was in line with procedures recommended by Nolte, McCaffery, and Snieder (6). Allele frequencies were calculated and analyzed for deviations from the Hardy-Weinberg equilibrium (HWE) using χ^2^-test (χ^2^ = 0.16, *p*. = .89).

*Analyses*

First, we examined the correlations between our main variables (see Table S1 in Text S1). Secondly, we conducted several multiple regression analyses to test our hypotheses (see Table S3-S5 in Text S1). We started testing to what extent genotype moderated the relation between aggression and popularity (Table S3 in Text S1). In this model we included the main effects of aggression and genotype (coded as two dummy variables for the AG allele and the GG allele with AA allele as reference category). Further, we included the interaction terms for aggression with the AG allele and with the GG allele, allowing to test its moderation effects relative to the AA allele. In the next model, we tested whether genotype also moderated the relation of having female friends with popularity, including main effects and interactions between genotype and female friends (Table S4 in Text S1). Finally, we tested the association between aggression and popularity for boys with a low number of female friends (*N* = 185) versus boys with a high number of female friends (*N* = 100). We used the mean of female friends as cut-off point. We tested this interaction using two two-way interactions owing to oversaturation of the regression model (Table S3 in Text S1).

In addition, we tested to what extent the G-allele also moderated the association of prosocial behavior with likeability (Table S5 in Text S1). *Likeability* was based on nominations adolescents received from their classmates on the question “Who are your best friends”. Scores were summed and standardized within class, yielding a proportion score, *M*(*SD*) = .21 (.13). Likeability was modestly correlated with popularity (*r* = .19, *p* < .001), demonstrating sufficient discriminant validity of likeability from popularity. *Prosocial behavior* was based on five items derived from the Youth Self Report (YSR) [7]. Sample items are “I enjoy being in the company of others”, “I enjoy making people laugh”, and “I enjoy helping others”. The internal consistency of the scale was satisfactory (α = .66), *M*(*SD*) = .27 (.30).

As can be seen from Figure S1, prosocial behavior contributed to likeability for both the carriers of the AG-allele and the GG allele. However, having the G-allele without prosociality was clearly associated with lower likeability. This fits with the ‘enhancer’ effect. If one lacks likable characteristics then drawing attention to the not-likable characteristics will lower likeability. This is an additional support for the genetic mechanism suggested in our study. In addition to the analyses for boys, we also conducted the same analyses for female adolescents from the TRAILS sample (*N* = 327). These analyses revealed that genotype did not moderate either the effect of aggression or prosocial behavior on popularity and likeability.

Table S1

*Correlations between variables*

|  |  | 1 | 2 | 3 | 4 | 5 |
| --- | --- | --- | --- | --- | --- | --- |
| 1 | Popularity | - |  |  |  |  |
| 2 | Likeability | .19* | - |  |  |  |
| 3 | Aggressive behavior | .14* | .02 | - |  |  |
| 4 | Prosocial behavior | .03 | .14* | -.20* | - |  |
| 5 | Female Friendships | .27* | .58* | -.03 | .12* | - |

**p* < .05

Table S2

Mean comparison between genotypes

|  | Mean (*SD*) | | | Differences  between genotypes |
| --- | --- | --- | --- | --- |
|  | AA  (*N* = 50) | AG  (*N* = 124) | GG  (*N* = 111) |  |
| Popularity | .09 (.14) | .09 (.12) | .11 (.15) | *F* (2, 282) = 0.97, *p* = .34 |
| Likeability | .23 (.14) | .21 (.13) | .20 (.12) | *F* (2, 282) = 0.68, *p* = .51 |
| Aggressive behavior | .27 (.37) | .27 (.27) | .28 (.29) | *F* (2, 282) = 0.06, *p* = .94 |
| Prosocial behavior | 1.50 (0.42) | 1.49 (0.41) | 1.53 (0.37) | *F* (2, 282) = 0.27, *p* = .77 |
| Female friendships | .07 (.10) | .08 (.13) | .05 (.09) | *F* (2, 282) = 1.62, *p* = .20 |

Table S3

*Standardized multiple regression coefficients* predicting popularity

|  | All Male Adolescents  (*N* = 285) | | Male Adolescents with Low Number of Female Friends  (*N* = 185) | | Male Adolescents with High Number of Female Friends  (*N* = 100) | |
| --- | --- | --- | --- | --- | --- | --- |
|  | B | *t* | B | *t* | B | *t* |
| AG | -.002 | -.02 | -.032 | -.31 | .035 | .27 |
| GG | -.077 | .94 | .018 | .17 | .236 | 1.77 |
| Aggressive behavior | -.112 | -1.00 | -.128 | -.91 | -.041 | .223 |
| AG x Aggressive behavior | .163 | 1.85+ | .118 | 1.08 | .209 | 1.37 |
| GG x Aggressive behavior | .249 | 2.76* | .289 | 2.51* | .349 | 2.47* |

Table S4

*Standardized multiple regression coefficients predicting popularity*

|  | All Male Adolescents  (*N* = 285) | |
| --- | --- | --- |
|  | B | *t* |
| AG | -.010 | -.13 |
| GG | .12 | 1.57 |
| Female friends | .16 | 1.13 |
| AG x Female friends | -.003 | -.023 |
| GG x Female friends | .246 | 2.69* |

Table S5

*Standardized multiple regression coefficients predicting likeability*

|  | All Male Adolescents  (*N* = 285) | |
| --- | --- | --- |
|  | B | *t* |
| AG | -.059 | -.72 |
| GG | -.103 | -1.25 |
| Prosocial behavior | -.083 | -.63 |
| AG x Prosocial behavior | .176 | 1.65 |
| GG x Prosocial behavior | .187 | 1.93* |

References

1. De Winter AF, Oldehinkel AJ, Veenstra R, Brunnekreef JA, Verhulst FC, Ormel J (2005) Evaluation of non-response bias in mental health determinants and outcomes in a large sample of pre-adolescents. Eur J Epidemiol 20:173-181.

2. Huisman M, Oldehinkel AJ, de Winter A, Minderaa RB, De Bildt A, Huizink AC, et al (2008) Cohort profile: The Dutch TRacking adolescents individual lives survey; TRAILS. Int J Epidemiol 37:1227-1235.

3. Dijkstra JK, Lindenberg S, Veenstra R (2008) Beyond the class norm: Bullying behavior of popular adolescents and its relation to peer acceptance and rejection. J Abnorm Child Psychol 36:1289-99.

4. Dijkstra JK, Lindenberg S, Verhulst FC, Ormel J, Veenstra R (2009) The relation between popularity and aggressive, destructive, and normbreaking behaviors: Moderating effects of athletic abilities, physical attractiveness, and prosociality. J Res Adolesc 19:401-413.

5. Miller S, Dykes D, Polesky H (1998) A simple salting out procedure for extracting dna from human nucleated cells. Nucleic Acids Res 16:1215.

6. Nolte IM, McGaffery JM, Snieder H (2009) Candidate gene and genome-wide association studies in behavioral medicine. In: Steptoe A, editor. Handbook of Behavioral Medicine: Methods and Applications. New York: Springer.

7. Achenbach TM (1991) Manual for the YSR and 1991 profile. Burlington VT: University of Vermont Department of Psychiatry.
